# Effects of Partial Substitution of Conventional Protein Sources with Duckweed (*Lemna minor*) Meal in the Feeding of Rainbow Trout (*Oncorhynchus mykiss*) on Growth Performances and the Quality Product

**DOI:** 10.3390/plants11091220

**Published:** 2022-04-30

**Authors:** Elisa Fiordelmondo, Simona Ceschin, Gian Enrico Magi, Francesca Mariotti, Nicolaia Iaffaldano, Livio Galosi, Alessandra Roncarati

**Affiliations:** 1 School of Biosciences and Veterinary Medicine, University of Camerino, Viale Circonvallazione 93–95, 62024 Matelica, Italy; elisa.fiordelmondo@unicam.it (E.F.); gianenrico.magi@unicam.it (G.E.M.); francesca.mariotti@unicam.it (F.M.); livio.galosi@unicam.it (L.G.); 2 Laboratory of Systematic and Environmental Botany, Department of Sciences, Roma Tre University, Viale G. Marconi 446, 00146 Rome, Italy; simona.ceschin@uniroma3.it; 3 Department of Agricultural, Environmental and Food Sciences, University of Molise, Via De Sanctis s/n, 86100 Campobasso, Italy; nicolaia@unimol.it

**Keywords:** sustainable fish feed, aquatic plants, alternative proteins, on-growing phase

## Abstract

Duckweed (*Lemna minor*) meal was included in the formulation of three experimental feeds (L1, L2, L3) for rainbow trout at 10%, 20%, 28% of the protein source, respectively. Increasing the duckweed inclusion, the other protein sources were adjusted to get isonitrogenous (41%) and isolipidic (20%) diets, as the control diet (LC). 540 fish (mean body weight 124.5 ± 0.7 g) were randomly allocated in 12 tanks divided equally among the four different diets. After 90 days, fish were weighed and the most important productive performances, fillet quality and fatty acid profile were determined. The final body weight in L1 (340.53 g) and L2 (339.42 g) was not different from LC (348.80 g); L3 trout significantly (*p* < 0.05) exhibited the lowest one (302.16 g). Similar trends were found in final mean length, weight gain, specific growth rate, food conversion rate. Somatic indices were affected by duckweed inclusion. Diets had not significant effects on the proximate composition and fatty acids of the fillet in L1, L2, L3 respect to LC. Based on this study, duckweed meal derived from *Lemna minor* can be included in the feed for the rainbow trout without negative effects on the growth performances at 20% of the protein substitution.

## 1. Introduction

According to Food and Agriculture Organization (FAO), fish consumption in the world has reached a new record of 20.5 kg per capita per year and it will increase further in the next decade [1]. In this context, sustainable development of aquaculture and effective management of fish resources are the key to support for this trend. The increase of fish production needs the use of new raw materials to be included in fish feeding, and the adoption of new technology and new strategies to produce greater quantities of fish in a sustainable way, thus avoiding natural resources exploitation. Nowadays, it is increasingly important to find alternative and innovative raw materials to be used in fish feeding, and to understand the level of the possible substitution without a negative influence in fish growth and fish meat quality, without forgetting the importance of the environmental sustainability. In particular, some non-conventional feeding sources are becoming strategically important from economic and environmental sustainability point of view; indeed, the use of expensive fish meal for fish feeding is leaving space for cheaper and less impactful alternative diets, based on protein sources able to replace this ingredient with others (insects, vegetables, algae, by-products from aquatic organisms) to combine fish growth and environmental sustainability [2,3]. The research of proteins of vegetable origin in the formulation of aquafeed has underlined that the crop-based agriculture can help the aquaculture to become more sustainable through expanding the variety of different plant sources [4,5]. The soybean meal is the conventional protein source mostly used as a complement to fish meal and consequently the demand for this feedstuff has significantly increased at the world level to skyrocket its price in these last five years; it is clear that this has made its use less and less sustainable both in economic terms, as many countries also have to import it, and in environmental terms. Therefore, the properties of other plants have been investigated to evaluate their potential use in fish feed as protein source [6,7,8]. Recently, the attention has been focused on small aquatic plants, known as duckweeds, appreciated for their ability to reduce nutrient concentrations in water absorbing nitrogen compounds [8,9] and for their nutritional properties [10].

Duckweeds (*Lemnaceae)* [11,12] are free-floating aquatic plants, occurring spontaneously in standing or slow-flowing waters. They grow very rapidly and widely in nature, showing to be one of the fastest growing higher plants [13,14], and their supply is easy to recover. These plants are characterized morphologically by a tiny (a few mm) leaf-shaped vegetative body (frond) in which the stem is not distinguishable from the leaves and with a root system consisting of a single root.

Concerning the cultivation, duckweeds can be produced quite easily and cheaply even without the necessity to use growth media and/or fertilizers since they are characterized by a high Relative Growth Rate (RGR) [14,15]. This means they are able to produce large quantities of biomass in a short time and in relatively small ponds filled with a few tens of centimeters of natural water (30–50 cm deep). Obviously, their productivity can increase the more the optimal ecological conditions for their growth are present. In optimal growth conditions, duckweeds show high concentrations of nutrients but pathogens, heavy metals and organic pollutants should be accumulated in the plants’ tissues [16]. Controlling and monitoring the aquatic environment in which the plants grow is particularly important. Duckweeds productivity increases more if the optimal ecological conditions for growth are respected, which however are generally wide. These, while varying slightly from species to species, generally consist of moderately warm, sunny and nutrient-rich waters, as documented in ecological studies on some duckweed species of the *Lemna* genus [12,17,18]. However, a good productive performance of duckweeds can occur in a wide range of conditions with respect to some factors, such as temperature and pH [7,8] for example, which clearly points out how these plants can be easily cultivable in different habitats. It is interesting to consider the hypothesis of growing duckweeds in wastewater from aquaculture systems [19] that, generally rich in nutrients, could allow a production of them at low cost and eco-sustainable in line with the principles of the circular economy. This is important when considering the huge quantity of plant needed in large scale fish feeding. Other aspects such as the rapid growth and the composition of protein and poly-unsaturated fatty acids [20,21] make duckweeds a good ingredient for feed applications.

Considering the composition of duckweeds, they provide a good source of protein (up to 45.5 g of crude protein [22]), lipid, and minerals [23,24,25]. For their good protein intake, duckweeds were also largely used for feeding ruminants [26,27], pigs [24,28] and poultry [29,30] and for making pet foods, as an alternative source of amino acids [20]. Duckweeds are also commonly consumed as food by people in some areas in different continents [31]. Amino acid profile and fatty acid profile have confirmed the suitability of these plants in the production of aquafeed [32]. Moreover, macronutrients and other compounds, such as β-carotene and xanthophyll, increase the importance of the duckweeds as a potential ingredient to be essayed in aquafeed [33] for warmwater fish species as rohu [34], carp [35] and tilapia [36]. However, in salmonids the dietary duckweeds meal content has been only evaluated during fry stage of the rainbow trout [22].

Based on these considerations, a trial was performed in order to evaluate the effects of duckweed meal as partial replacement of the main conventional protein sources (fish and soybean meal) in three different low fish meal diets on productive performances of rainbow trout reared during the on-growing phase and compared with coetaneous fish receiving a conventional feed.

## 2. Results

### 2.1. Water Physico-Chemical Characterization

Concerning the physico-chemical characteristics of the waters where the fish were reared, in all the groups the temperature ranged from 12 °C, at the beginning of the experiment, to 13.8 °C (L2) at the end (mean values: LC 11.05 ± 0.8 °C; L1 11.06 ± 0.9 °C; L2 11.04 ± 0.9 °C; L3 11.05 ± 0.9 °C). The pH ranged between 7.8 and 8.0 without notable variations among the groups (mean values: 7.9 ± 0.1). The dissolved oxygen was averagely always over 10 mg/L in all the groups (mean values: LC 10.9 ± 1.5 mg/L; L1 10.8 ± 1.6 mg/L; L2 10.9 ± 1.4 mg/L; L3 10.2 ± 1.2 mg/L). Water total nitrogen ammonia (TAN) was included between a minimum in LC and L3 tanks (0.11–0.12 mg/L) and a maximum in L2 and L3 (0.19–0.20 mg/L) (mean values: LC 0.16 ± 0.1 mg/L; L1 0.15 ± 0.05 mg/L; L2 0.15 ± 0.06 mg/L; L3 0.16 ± 0.06 mg/L). Nitrites (NO_2_-N) ranged from 0.02 mg/L in LC to 0.03 mg/L in L3 tanks (mean values: LC 0.025 ± 0.004 mg/L; L1 0.029± 0.002 mg/L; L2 0.026 ± 0.003 mg/L; L3 0.028 ± 0.002 mg/L) while nitrates (NO_3_-N) from 0.9 mg/L (L2) to 0.14 mg/L (L3) (mean values: LC 0.12 ± 0.2 mg/L; L1 0.12 ± 0.1 mg/L; L2 0.10 ± 0.1 mg/L; L3 0.12 ± 0.2 mg/L).

### 2.2. Productive Performances of Oncorhynchus mykiss under Different Experimental Diets

The productive parameters are reported in Table 1. The final mean body weight and length did not show significance differences among trout fed with L1 (340.5 g; 31.2 cm), L2 (339.4 g; 31.6 cm) and LC group (348.8 g; 31 cm) but were different from L3 group (302.16 g; 28.2 cm) that showed the lowest significantly final weight and size. Growth parameters, weight gain (WG) and specific growth rate (SGR), recorded similar performances among L1 (216 g; 1.26), L2 (214.9; 1.2) and LC (224.3; 1.29) and were significantly higher than L3 (177.66; 1.11). Food conversion rate (FCR) gave a favorable result without statistically differences among L1 (1.18), L2 (1.18) and LC (1.13) but significantly better than the one recorded in L3 group (1.37).

The somatic indices, condition index (KI), viscerosomatic index (VSI), perivisceral fat index (PFI), hepatosomatic index (HSI), were significantly affected by diets with different levels of duckweed meal. KI exhibited differences when the inclusion was at the highest substitution (28%) in L3 group (1.35) respect to all the other groups (L1: 1.12; L2: 1.08; LC 1.17). VSI significantly increased from L1 (10.3), at an intermediate level between LC (10.06) and L2 (11.57), to L3 (14.57). PFI had not notable variations among L1 (2.9), L2 (3.05) and LC (3), that were all different from L3 (3.68). HSI appeared similar among the three experimental groups (L1: 1.31; L2: 1.35; L3: 1.24) but significantly higher than LC (1.05). No significant differences were observed in the survival rate (SR), with values ranging between 98 and 99% in all groups. Palatability of L1 (99.6) was at an intermediate level respect to LC (100) and L2 (98.8) and L3 (98.2); these last two experimental diets showed a similar acceptance.

The proximate composition of the fillets of rainbow trout fed with diets without (LC) or with different percentages of duckweed meal (L1, L2, L3) is reported in Table 2. For all the macronutrients considered (protein, fat, moisture and ash content) no significant differences were shown among the duckweed meal diets (L1, L2, L3) and the conventional control diet (LC).

**Figure 1:**
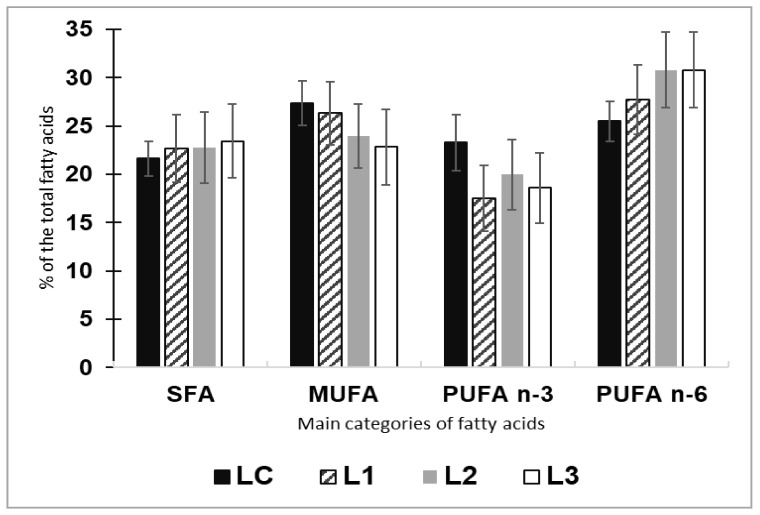
Categories of fatty acids (SFA: saturated fatty acids, MUFA: monounsaturated fatty acids, PUFA: n-3, n-6 polyunsaturated fatty acids) determined in the fillet of trout fed with LC and L1, L2, L3 diets (% of the total fatty acids). Due to no significant difference from the statistical point of view, no letter was reported in the groups of column charts.

With regard to the main categories of fatty acids in the final composition of the fillet of rainbow trout, the saturated fatty acids (SFA) and monounsaturated fatty acids (MUFA) categories were similar among all the four groups without notable differences (Figure 1). Furthermore, the polyunsaturated fatty acids (PUFAs) (n-3 and n-6 series) did not show significant differences (*p* < 0.05) between the experimental (L1, L2, L3) and the control (LC) group (Figure 1).

## 3. Discussion

For decades, duckweed has captured the interest of scientists [37] due to its organoleptic characteristics. It is still a current topic due to the urgent problem of finding new protein sources as alternatives to the standard ones to be used in aquafeed. In particular, the properties of duckweed meal have been studied for their protein content in freshwater and marine fish.

In this trial, duckweed meal was included in three experimental diets for rainbow trout as partial replacement of the two main protein feedstuffs, fish meal and soybean meal, in order to evaluate an alternative protein source less expensive and more sustainable than conventional ones. The substitution has mostly concerned soybean meal and secondly fish meal, because in the last years the strong increase of the production costs of aquafeeds has also concerned the price of feedstuffs of vegetable origin such as soybean meal. Besides, the maintenance of a fraction of fish meal was considered prudent by authors to assure good growth results for rainbow trout. To perform the study, we used duckweed collected in freshwater areas, strictly monitored in terms of quality as demonstrated by the analyzed physico-chemical parameters were within the range considered optimal for the rainbow trout species in all the fish groups tested [38]. The survival rate was very high in all the experimental groups.

The meal obtained by the common duckweed employed in the feeds had a protein content within the range specifically reported for that plant species [36]. The amount of essential amino acids was detected in a good quantity, except methionine. Because of that, DL-methionine was added in the formulation of all the three experimental diets used in the present trial, with the aim to administer balanced diets according to the specific requirements of the rainbow trout species [39]. The percentages of duckweed substitution were also evaluated and decided making a gradual increase of duckweed in the experimental diets. In duckweed meals used in the present study, the fat content (5%) was in agreement with the literature reporting ranges between 4% and 7% [24].

The productive results showed that no adverse effects were observed in mean body weight, weight gain, and final length of rainbow trout when fish receives diets including up to 20% of duckweed meal, whereas highest levels affected the growth performances and the FCR. These differences could be related to the diet palatability that slightly decreased at increasing the duckweed meal. As Table 1 shows, the decrease of the palatability of the duckweed meals corresponds to an up inclusion of *Lemna* meal. The rainbow trout receiving the feed with the highest duckweed meal content exhibited reduced productive performance and unfavorable FCR. In another study on the taste of various aquatic plants for tilapia [40], duckweed showed a low attractive effect due to the presence of flavonoids and triterpene compounds considered as not feed stimulating for fish. However, in common carp, diets with 20% of duckweed replacement gave results similar to conventional feeds in terms of growth performances [34]. In the Indian carp species (rohu *Labeo rohita*), the replacement of 30% of fish meal dietary with common duckweed did not affect the growth of fingerlings in comparison with coetaneous fed with other macrophytes [41]. Based on literature, studies on common duckweed in fish diets were mostly performed on cyprinids and tilapia, so fish with feeding (omnivorous) and living (warmwater) habits different respect to salmonids. In rainbow trout, a 4 weeks trial using another species of duckweed (*Spirodela polyrhiza*) at low and high (6.25–12.5% of feed) substitution for 4 weeks, had the same acceptance of the control diet although both the duckweed meal treatments resulted in 5 and 10% poorer growth traits [22]. As concerns the somatic indices, KI and PFI were affected by the highest percentage of duckweed inclusion followed the trend of growth increased, whereas VSI and HSI discriminated differences also among the three experimental diets as well as with the control feed. These variations in VSI and HSI could be associated to the carbohydrate fraction not used as energy source, and therefore accumulated in the liver and transformed in lipids and glycogen, resulting in an increase of this index as documented in rainbow trout fed with diets including alternative plant ingredients rich in indigestible carbohydrates, in the form of oligo- as well as polysaccharides [42]. To overcome this drawback, other works proposed to employ duckweed after a fermentation process that could considerably reduce the antinutritional factors and the crude fiber content [28]. Regarding the effects of dietary duckweed on the fish fillet quality, the proximate composition did not show notable differences among the macronutrients of the groups. In fact, as reported in Table 2, the proximate composition of the fillet of rainbow trout fed with LC, L1, L2 and L3 doesn’t show significant differences from the statistical point of view. In terms of fatty acid categories in the meat, trout fed diets including *Lemna* appeared very similar among them and the control. It is well known that the final nutritional flesh quality is strongly affected by the diet composition administered to fish [43]. In the current study, the experimental feeds maintained the blend of fish and vegetable oil unchanged respect to the control diet with the aim to show the only effects of the duckweed meal ingredient. According to a study on nutritional value of different duckweed genera, *Lemna minor* is reported as a species with an intermediate proportion (27.99%) of SFA, a very low MUFA level (4.6%) and a very high PUFA n-3 rate (46%), surprisingly higher than content of PUFA n-6 (20%) [21]. In the current experiment, the fillet of rainbow trout had good content of fatty acids especially in terms of PUFA n-3, considered essentials of a balanced diet in humans providing beneficial effects on neural development [44] and in mitigating several pathological conditions [45].

## 4. Materials and Methods

### 4.1. Experimental Design and Fish Material

For the experiment, 12 concrete outdoor tanks were used, divided in 3 tanks for each of the four trout groups fed with different diets (LC, L1, L2, L3). Every tank had a length of 5 m, a width of 0.8 m, a depth of 0.5 m, and a volume of 2 m^3^ and was filled with well water. During the experiment, the main water physico-chemical parameters (temperature, dissolved oxygen and pH) were daily recorded in every tank using portable electronic devices (YSI mod. 55 and 60, Yellow Springs, OH, USA). TAN, NO_2_-N and NO_3_-N were weekly analyzed following APHA standard methods [46]. All the basins were covered with an antifouling net in order to avoid algal development and to keep away ichthyophagous birds.

This experiment was performed during a standard zootechnical cycle, avoiding any animal suffering, and no sample was collected from live animals, according to the Italian Legislative Decree 26/2014. The farm applied an “Antibiotic-free Code of Prescription” to guarantee the quality of the product and respect the “antibiotic-free” approach. During spring season in 2021, a total of 540 rainbow trout (245 days old; mean body weight 124.5 ± 0.7 g) was randomly allocated in the 12 tanks, with 45 fish in each tank, at the initial stocking density of 6.2 kg/m^3^. At the end of the experiment (90 days), fish were weighed, and their final length was recorded. Fish were fed by hand twice a day (8 a.m. and 3 p.m.) until the apparent satiation level; then the unconsumed feed was collected.

Palatability of the feeds was calculated according to the formula: ((ingested feed/administered feed) × 100) based on the index reported in previous studies [47,48]. The following zootechnical performances were evaluated in the four different groups: WG (%) = (final weight − initial weight) × 100 ∕initial weight; SGR (%∕day) = {Ln (final weight) − Ln (initial weight) ∕days} × 100; FCR = live weight gain (g)/feed administered (g); SR (%) = final number of fish/initial number of fish × 100 [49]. In addition, the condition factor KI = ([fish weight/fish length] × 100) [50] and the following biometric indices were calculated after having applied standard procedures for fish sampling [51]: VSI = ([weight viscera/whole body weight] × 100), PFI = ([perivisceral fat/body weight] × 100), and HSI = ([liver weight/body weight] × 100). In order to calculate the PFI and the VSI, the fat adherent to the digestive tract was accurately separated and individually weighed.

### 4.2. Plant Material and Experimental Diets

Fronds of a duckweed species of the genus *Lemna*, *L. minor* (common duckweed) were collected from ponds of a fish farm. We avoid the use of duckweeds coming from polluted areas or wastewater due to high sensitivity of this plant to a wide range of toxicants [52] which could threaten the fish health status [40]. Samples of duckweed were analyzed from the botanical point of view by means of stereomicroscope (mod. Stemi 305, Zeiss). The duckweed was submitted to washing, air exposure and oven-dried at 60 °C for 6 h, and finally milled modified after recent literature [30]. In order to employ the duckweed meal for the on-growing phase of rainbow trout, the proximate composition and the amino acid profile were determined on a duckweed frond sample (Table 3). The proximate composition (moisture, protein, lipid and ash) and the amino acid profile of duckweed meal were performed according to international methods. In particular, moisture and ash content were determined using the procedures described by the Official Analytical Chemists (AOAC) [53]. The protein content was determined using the standard Kjeldahl copper catalyst method. The aminoacid profile was determined by acid hydrolysis (6 N HCl for 24 hrs, at 110 °C) followed by ion ex-change chromatography with an amino-analyzer (L-8800 Auto-analyzer, HITACHI, Japan).

*Lemna minor* meal was included in the formulation of three feeds (L1, L2, L3) at different rates (10%, 20%, 28%, respectively) of the protein source. At the increasing of the duckweed inclusion, soybean meal, fish meal, wheat flour and gluten wheat meal were reduced or adjusted in order to get similarly an isonitrogenous (41%) and isolipidic (20%) diets. A control diet (LC) was formulated with the same feedstuffs except duckweed meal (Table 4). This formulation aimed at saving the use of the various conventional protein sources, essaying a local feedstuff derived from duckweed plant; after the pandemic, also soybean meal and not only fish meal are becoming very expensive to get on the market.

The feeds were manufactured in 3.5 mm size using a twin-screw extruder (100 rpm, 110 °C, 50 atm). After the coating, the feeds were stocked in buckets and kept in an aerated room. The proximate composition (moisture, protein, lipid and ash) and the amino acid profile of three samples of each feed type were performed according to the international methods reported for duckweed meal analysis [53]. For all the four diets, after determining total lipid content, using the procedure described by Folch et al. [54], fatty acids were converted to methyl esters following the method described by Christopherson and Glass [55]. The separation of fatty acids was carried out using a GC 3800 gas chromatography (Varian Strumentazione, Cernusco sul Naviglio, Italy) with a WP-4 Shimadzu integration system (Shimadzu Corporation, Tokyo, Japan), which was equipped with a Supelco SPTM—2340 capillary column (30 m × 0.25 mm i.d.; 0.25 μm film thickness; Supelco, Bellefonte, Pennsylvania, USA) and a flame ionization detector. The essential amino acid profile of the three feeds containing duckweed was obtained as previously indicated for duckweed meal.

### 4.3. Quality Traits of Fish Fillet

After 90 days of experiment, fish have reached the commercial size and are slaughtered by electrical stunning in an authorized slaughterhouse.

Fish were dissected as follow: after a check of the skin status on the whole body to be sure of the absence of skin lesions, the abdomen was opened with a cut starting from the anus to gills, and then a lateral line up the side of the fish allowed to check the status of gills and meat. From the anus the digestive system was tracked and removed; similarly, all the abdomen organs were checked and removed.

A portion of about 50 g of skinless dorsal left muscle from six fish casually selected for each diet group was collected, then homogenized and submitted to proximate composition analyses (moisture, protein, lipid, and ash content). The procedures adopted for these last analyses follow substantially the same methods indicated in the previous paragraph concerning the measurement of proximate composition of the different feedstuffs. The percentage of moisture was determined in duplicate according to the Association of Official Analytical Chemists procedure [53]. The protein content was determined using the standard Kjeldahl copper catalyst method. The ash content was determined using the procedure described by the AOAC [53]. Total lipids were measured using a modification of the chloroform:methanol procedure described by Folch et al. [54]. After determining the total lipid content, fatty acids were converted to methyl esters following the method described by Christopherson and Glass [55]. The separation of fatty acids was performed using a Carlo Erba HRGC 5160 gas chromatography (Carlo Erba Strumentazione, Rodano, MI, Italy) with a WP-4 Shimadzu integration system (Shimadzu Corporation, Tokyo, Japan) equipped with a Supelco SPTM-2340 capillary column (30 m× 0.32 mmi.d.; 0.20 μm film thickness; Supelco, Bellefonte, PA, USA) and a flame ionization detector. The operating conditions of the gas chromatography were as follows: the oven temperature was set at 170 °C for 15 min and subsequently increased to 190 °C at a rate of 1 °C/min, then increased to 220 °C at a rate of 5 °C/min and held at this temperature for 17 min. The concentration of individual fatty acid was calculated based on the relative proportion of each fatty acid compared with a known amount of the internal standard (17:0) added. The fatty acids were expressed as percentage of the total of fatty acids.

### 4.4. Statistical Data Analyses

Data collected (biometric parameters, final productive traits, fish fillet proximate composition, fatty acids categories) were subjected to one-way analysis of variance (ANOVA) using SPSS 25 [56] to check differences in productive performances and composition of fillet of rainbow trout fed with different experimental diets. Means and standard deviations were calculated. Means were considered significant with a value of *p* < 0.05 and compared using the Student-Newman-Keuls (SNK) test.

## 5. Conclusions

The current study aimed at evaluating the use of a local duckweed, collected in not contaminated waters, as protein source in partial substitution of the conventional feedstuffs (fish meal and soybean meal) to find out how could be used in animal feeding respecting the nature of the animal species. In particular, it provided useful information on the effects of duckweed meal diet on rainbow trout performances under on-growing phase and the quality fillet. This has great implication on responsible and sustainable aquaculture because it is essential to preserve fish biometric indices, rearing parameters and quality product, mainly when fish are reared under antibiotic-free approach. Through this study it has been possible to essay a protein source substitution with an alternative plant feedstuff, showing that the replacement should be done using *Lemna minor* at 20% of the protein sources, without negative consequences in the growth performance of the fish and quality fillet. At this rate of protein content, the feed with duckweed meal has shown satisfactory results. In this view, such study can represent a challenge for further investigations aimed to analyze possible variations of duckweed composition in different seasons, in different areas or considering different *Lemna* species occurring locally.

## Figures and Tables

**Table 1:** Productive performances of rainbow trout fed with different experimental diets (mean ± standard deviation).

Parameters	LC	L1	L2	L3	*p*
Initial mean weight (g)	124.5 ± 0.7	124.5 ± 0.7	124.5 ± 0.7	124.5 ± 0.7	-
Initial mean length (cm)	20.0 ± 0.6	20.0 ± 0.6	20.0 ± 0.6	20.0 ± 0.6	-
Final mean weight (g)	348.80 ± 4.4 a	340.53 ± 4.3 a	339.42 ± 4.7 a	302.16 ± 2.2 b	<0.05
Final mean length (cm)	31.0 ± 1.2 a	31.2 ± 1.3 a	31.6 ± 1.5 a	28.2 ± 1.6 b	<0.05
WG (%)	224.3 ± 2.6 a	216.03 ± 2.8 a	214.92 ± 2.9 a	177.66 ± 2.7 b	<0.05
SGR (%/day)	1.29 ± 0.03 a	1.26 ± 0.04 a	1.25 ± 0.03 a	1.11 ± 0.01 b	<0.05
FCR (g/g)	1.13 ± 0.02 b	1.18 ± 0.02 b	1.18 ± 0.03 b	1.37 ± 0.02 a	<0.05
SR (%)	99 ± 0 a	98 ± 1 a	98 ± 1 a	98 ± 1 a	<0.05
Palatability	100 ± 0.0 a	99.6 ± 0.4 ab	98.8 ± 1 b	98.2 ± 1.1 b	<0.05
KI	1.17 ± 0.12 a	1.12 ± 0.13 a	1.08 ± 0.14 a	1.35 ± 0.22 b	<0.05
VSI	10.06 ± 0.41 c	10.28 ± 0.59 bc	11.57 ± 0.68 b	14.57 ± 0.54 a	<0.05
PFI	3.00 ± 0.36 b	2.91 ± 0.04 b	3.05 ± 0.12 b	3.68 ± 0.03 a	<0.05
HSI	1.05 ± 0.06 b	1.31 ± 0.08 a	1.35 ± 0.03 a	1.24 ± 0.06 a	<0.05

Different letters (a, b, c) on the same line show statistically significant differences (*p* < 0.05). WG: weight gain, SGR: specific growth rate, FCR: feed conversion rate, SR: survival rate, KI: condition index, VSI: viscerosomatic index, PFI: perivisceral fat index, HSI: hepato-somatic index.

**Table 2:** Proximate composition (% ww) (mean ± st.dev.) of the fillet of rainbow trout fed with the control diet (LC) and the three experimental diets (L1, L2, L3) at the end of the trial.

Parameters	LC	L1	L2	L3	*p*
Moisture	77.73 ± 1.4	77.76 ± 1.2	77.35 ± 1.1	77.41 ± 1.2	>0.05
Protein	19.44 ± 0.9	19.78 ± 1.0	18.46 ± 1.1	18.82 ± 0.8	>0.05
Fat	2.33 ± 1.1	2.54 ± 0.8	3.17 ± 0.9	3.31 ± 0.9	>0.05
Ash	1.37 ± 0.2	1.29 ± 0.1	1.25 ± 0.2	1.18 ± 0.2	>0.05

Due to no significant differences from the statistical point of view, no letter was reported among the parameters on the same line.

**Table 3:** Proximate composition (%) and amino acid profile (g/100 g) of duckweed meal used in the experiment.

Composition	%
Moisture	92.81
Crude protein	28.13
Crude lipid	5.10
Crude fibre	15.20
Ash	16.40
*Amino acid*	% of crude protein
Arginine	4.56
Histidine	3.28
Isoleucine	3.62
Leucine	6.41
Lysine	4.49
Methionine	1.74
Phenylalanine	4.25
Threonine	2.16
Tryptophan	3.89
Valine	3.53

**Table 4:** Formulation and proximate composition of the control diet without including duckweed meal (LC), and L1, L2 and L3 diets, with different inclusion of duckweed meal (10%, 20%, 28%, respectively).

	LC	L1	L2	L3
*Ingredients (%)*				
**Duckweed meal**	**0**	**10**	**20**	**28**
Soybean meal	22.3	21.8	11.0	7.0
Fish meal	21	20	18	17
Wheat flour	20	13	11	8
Haemoglobin meal	10	10	10	10
Gluten wheat meal	7.6	5.6	10.4	11.3
Fish oil	12	12	12	12
Soybean oil	5	5	5	5
L-Lysine	0.4	0.4	0.4	0.4
DL-Methionine	0.18	0.2	0.25	0.28
Vitamin-mineral mix	2	2	2	2
*Proximate composition (%)*				
Moisture	8.89	8.92	8.97	9.05
Protein	41.50	41.59	41.53	41.27
Lipid	20.00	20.12	20.30	20.00
Ash	6.81	7.28	7.10	7.26
*Amino acid profile (% of crude protein)*				
Arginine	5.24	4.51	4.00	3.92
Histidine	1.85	1.68	1.67	1.50
Isoleucine	1.39	1.37	1.31	1.30
Leucine	3.61	3.60	3.54	3.51
Lysine	4.99	3.90	3.56	3.01
Methionine	3.33	2.91	2.91	2.91
Phenylalanine	2.28	2.10	1.94	1.80
Threonine	2.06	1.53	1.45	1.42
Tryptophan	0.60	0.50	0.49	0.47
Valine	3.36	2.36	2.31	2.28

## Data Availability

Data is contained within the current article.
